# Detection of alien genetic introgressions in bread wheat using dot-blot genomic hybridisation

**DOI:** 10.1007/s11032-017-0629-5

**Published:** 2017-03-09

**Authors:** María -Dolores Rey, Pilar Prieto

**Affiliations:** 1 Plant Breeding Department, Institute for Sustainable Agriculture, Agencia Estatal Consejo Superior de Investigaciones Científicas (CSIC), Alameda del Obispo s/n, 14080 Córdoba, Spain; 2 Crop Genetics Department, John Innes Centre, Norwich, NR4 7UH UK

**Keywords:** Dot-blot hybridisation, Genomic introgressions, *H. chilense*, Bread wheat

## Abstract

Simple, reliable methods for the identification of alien genetic introgressions are required in plant breeding programmes. The use of genomic dot-blot hybridisation allows the detection of small *Hordeum chilense* genomic introgressions in the descendants of genetic crosses between wheat and *H. chilense* addition or substitution lines in wheat when molecular markers are difficult to use. Based on genomic in situ hybridisation, DNA samples from wheat lines carrying putatively *H. chilense* introgressions were immobilised on a membrane, blocked with wheat genomic DNA and hybridised with biotin-labelled *H. chilense* genomic DNA as a probe. This dot-blot screening reduced the number of plants necessary to be analysed by molecular markers or in situ hybridisation, saving time and money. The technique was sensitive enough to detect a minimum of 5 ng of total genomic DNA immobilised on the membrane or about 1/420 dilution of *H. chilense* genomic DNA in the wheat background. The robustness of the technique was verified by in situ hybridisation. In addition, the detection of other wheat relative species such as *Hordeum vulgare*, *Secale cereale* and *Agropyron cristatum* in the wheat background was also reported*.*

## Introduction

The introgression of genetic material from wild or distantly related species into wheat germplasm is a classical and effective approach for broadening the genetic basis of this crop. Hybridisation with bread wheat-related species makes it possible to transfer agronomically useful genes from those relative species into the wheat background. For example, wheat is affected by several fungal diseases; biototrophic fungi cause leaf and stripe rust diseases, as do powdery mildew or necrotrophic fungi such as *Septoria tritici* and *Fusarium graminearum* (Duveiller et al. 2007). Many relatives such as *Hordeum* species can be used as genetic tools to transfer resistance genes for these diseases into wheat. For example, there are genes conferring resistance to powdery mildew on *Hordeum vulgare* chromosome 1**H** ^**v**^ (Graner et al. 1991), genes conferring resistance to *Puccinia graminis* on *H. vulgare* chromosomes 5**H** ^**v**^ and 7**H** ^**v**^ (Kleinhofs et al. 1993; Borovkova et al. 1995), genes conferring resistance to *Septoria tritice* on *H. chilense* chromosome 4**H** ^**ch**^ (Rubiales et al. 2000) and tolerance to greenbug (*Schizaphis graminum* Rond.) on chromosome 5**H** ^**ch**^ from *H. chilense* (Castro et al. 2011). *Hordeum chilense* chromosome addition and substitution lines were developed in bread wheat and used for the transfer of wild barley genes into wheat (Miller et al. 1982). Similar cytogenetic stocks have been developed involving the cultivated barley *H. vulgare* (Islam et al. 1978, 1981; Szakács and Molnár-Láng 2006; Molnár-Láng et al. 2012) and rye (*Secale cereale* L.) chromosomes (Chapman and Riley 1955; Riley and Chapman 1958a; Miller 1984).

Breeders use genetic crosses to introduce into crops desirable genes from exotic germplasms, but sexual hybridisation between polyploid wheat and wild species generally produces an interspecific hybrid containing a haploid set of polyploid and wild relative chromosomes. In many such hybrids, there is only a low level of pairing and recombination between wheat and wild relative chromosomes. This failure of homoeologous (related) pairing during meiosis between wheat chromosomes and those from the related species is mainly due to the *Ph1* locus (Okamoto 1957; Riley and Chapman 1958b; Sears and Okamoto 1958; Sears 1977). Since the characterisation of the *Ph1* locus, its absence (*ph1b* mutant) has been used widely and successfully in wheat to induce homoeologous pairing and recombination (Sears 1977, 1981, 1982; Riley et al. 1968; Lukaszewki, 2000; Qi et al. 2008; Liu et al. 2011; Zhao et al. 2013). In the absence of the *Ph1* locus, all chromosomes can remodel without requiring the presence of identical or near-identical chromosomes, and this increases the chance of pairing between related and wheat chromosomes (Prieto et al. 2004a; Lukaszewki 2000; Rey et al. 2015). In fact, the use of the *ph1b* mutant allowed the linkage drag of the relative species in the wheat background to reduce and obtained recombinants between those relatives and bread wheat (Lukaszewki 2000; Rey et al. 2015). Thus, recombination can be possible between related chromosomes using the *ph1b* mutant, although crossovers still occur randomly between homoeologues from both species. Other methods have been used in breeding to introgress desirable characters from related species into bread wheat. For example, ionising radiation has been applied to induce random chromosome breaks to transfer resistance genes from *Aegilops umbellulata* Zhuk., *Agropyron intermedium*, *Agropyron elongatum* or rye into wheat (Sears 1956; Friebe et al. 1993, 1995). Wheat-barley translocation lines have been also obtained by using gametocidal genes (Gc genes) of *Aegilops cylindrical* Host. (Endo et al. 1998) and derivatives of hybrids multiplied in vitro (Molnár-Láng et al. 2000). However, all these methods are random and the linkage drag is not reduced.

The screening and identification of alien genomic introgressions in the descendence of interspecific genetic crosses can be difficult, especially when chromosome pairing and recombination occur randomly and the alien genomic introgressions have been obtained arbitrarily in the background of a crop species. Particularly in wheat, this is also limited by the complexity of its genome and the high level of synteny among wheat and related species (Moore et al. 1995; Salse and Feuillet 2007). The use of molecular markers combined with in situ hybridisation is very useful for finding exogenous genetic introgressions (Schwarzacher et al. 1989; Calderón et al. 2012; Zhao et al. 2013), but the exogenous chromosome fragment needs to be previously identified and well characterised in order to choose specific molecular markers that will allow the alien sequence to be unequivocally distinguished from the equivalent chromosome region in wheat, which can sometimes be difficult. In addition, in situ hybridisation enables the determination of the exact chromosomal compositions in the descendence of genetic crosses between wheat and related species (Prieto et al. 2001). However, although in situ hybridisation is an enormously informative genetic approach, it requires high expertise and is time consuming, making the cytogenetic approach expensive when there is a need to analyse hundreds of plants resulting from genetic crosses. Thus, breeders must be provided with reliable and user-friendly methods of rapid assessment that can be routinely applied when large numbers of plants have to be screened.

Although dot-blotting is a simple method and expected to be suitable for analyses of large numbers of samples with low cost, it has not been adopted much in plant genome studies. Dot-blot hybridisation has been used since the 1980s as a routine assay to detect, for example, RNA sequences from small cultured cell samples (Cheley and Anderson 1984) or the presence of viruses in human tissues (Achim et al. 1994) and to measure the telomere DNA content (Kimura and Aviv 2011). This technique is extensively used in plants to detect viruses or pathogen infections (Owens and Diener 1981; Liu et al. 2007; Vassilakos et al. 2012; Azza and Eman 2016), evaluate intergeneric *Saccharum* × *Erianthus* hybrids (Besse et al. 1997) and for the identification of species in the tribe *Brassiceae* using repetitive DNA sequences (Tonosaki and Nishio 2010), among other numerous examples. In the present study, we have adapted and optimised the genomic dot-blot hybridisation technique to be used as a routine and low-cost tool to rapidly screen a large population of plants carrying small random chromosome introgressions from *H. chilense* in the wheat background in a breeding programme framework. In addition, the technique was also tested for other wheat relative species that are also used in wheat breeding programmes. The high accuracy and feasibility of the genomic dot-blot technique to analyse many individuals could facilitate the screening and selection of plants carrying alien genetic introgressions in a crop breeding programme.

## Materials and methods

### Plant material

The plant material used in this work included the wild barley *H. chilense* Roem. et Schult., wheat lines (*T. aestivum* cv. *Chinese Spring*) carrying either one or two full copies of a *H. chilense* chromosome (monosomic or disomic *H. chilense* addition lines; 2*n* = 6*x* + 1 = 43 and 2*n* = 6*x* + 2 = 44, respectively), wheat lines having one or two copies of a telosomic *H. chilense* chromosome (monotelosomic and ditelosomic *H. chilense* addition lines; 2*n* = 6*x* + 1*t* = 42 + 1*t* and 2*n* = 6*x* + 2*t* = 42 + 2*t*, respectively) and wheat lines carrying a copy of a distal introgression of chromosome 4**H** ^**ch**^ from *H. chilense* (approximately 1/10 of the total chromosome length) which is about a 1/420 dilution of *H. chilense* DNA in the wheat background . In addition, *H. vulgare*, *S. cereale* and *Agropyron cristatum* species were also included in this work. All the lines were kindly supplied by Dr. Steve Reader (JIC, Norwich, UK) except the wheat line carrying the distal *H. chilense* introgression of chromosome 4**H** ^**ch**^, which was developed in our lab (Rey et al. 2015).

### Dot-blot genomic hybridisation

The total genomic DNA was extracted from frozen seedling leaves following the Murray and Thompson (1980) procedure and modified by Hernández et al. (2001). The quality and the concentration of the DNA were verified by electrophoresis in 1% agarose gel. Genomic DNA samples (200 ng) were blotted onto nylon membranes (Hybond N^+^, Amersham International, Buckinghamshire, UK) and were prehybridised for 30 min at 75 °C in 50% formamide, 2× saline-sodium citrate (SSC), 0.1% sodium dodecyl sulphate (SDS) and 2% blocking reagent (Roche Diagnostics, Meylan, France) with gentle shaking. The hybridisation mixture, consisting of 50% formamide, 2× SSC, 0.1% SDS and 600 ng of biotin-genomic DNA probe (*H. chilense*, *H. vulgare*, *S. cereale* or *A. cristatum*, depending on the experiment), was added to the prehybridisation buffer. The total genomic DNA used as a probe was labelled by nick translation with biotin-11-dUTP (Boehringer Mannheim Biochemicals, Germany). The total wheat genomic DNA was also denatured at 99 °C for 1 h in a DNA thermal cycler (Veriti™ Thermal Cycler, Thermo Fisher Scientific, Somerset, New Jersey, USA) to allow fragmentation into pieces of 100–200 bp in size and employed as blocking DNA in the hybridisation mixture. *H. chilense* DNA probe and wheat blocking DNA were used in a 1:300 ratio in the hybridisation mixture. Hybridisation was conducted at 75 °C for 8 min followed by an overnight incubation at 37 °C. After hybridisation, the membrane was incubated in a Petri dish (9 cm diameter) with 100 mM Tris-HCl (pH 7.5) and 15 mM NaCl (buffer 1) for 1 min, followed by incubation in a blocking buffer (0.5% (*w*/*v*) blocking reagent from Roche Diagnostics Meylan, France) diluted in 100 mM Tris-HCl (pH 7.5) and 15 mM NaCl (buffer 2) for 30 min, shaking gently. The membrane was incubated with the antibiotin IgG Fab fragment conjugated with alkaline phosphatase (MACS, Bergisch Gladbach, Germany) diluted 1:100 in buffer 1 at 37 °C for 30 min, shaking gently. After the antibody incubation, the membrane was washed in buffer 1 for 15 min and then transferred to the detection buffer (buffer 3, 100 mM Tris-HCl, pH 9.5; 100 mM NaCl; 50 mM MgCl_2_) for 2 min. Finally, the hybridisation signals were developed by adding NBT (4-nitroblue tetrazolium chloride, 70% dimethylformamide; Sigma, St. Louis, MO, USA) and BCIP (5-bromo-4-chloro-2-indolylphosphate, 50 mg/ml in 70% dimethylformamide; Sigma, St. Louis, MO, USA) for 3 min in buffer 3 in the dark until the colour was fully developed. The membrane was then washed in distilled water and air-dried.

### Genomic in situ hybridisation

Total *H. chilense* genomic DNA was also labelled by nick-translation with biotin-11-dUTP (Boehringer Mannheim Biochemicals, Germany) or digoxigenin-11-dUTP (Roche Applied Science, Indianapolis, IN, USA) and used as a probe. The in situ hybridisation protocol was performed according to Prieto et al. (2004b). The amount of either the biotin- or digoxigenin-labelled probes in the hybridisation mixture was 5 ng. Unlabelled wheat genomic DNA was used as blocking DNA at a ratio of 1:50 (probe/blocking DNA). Biotin-labelled *H. chilense* DNA and digoxigenin-labelled *H. chilense* DNA were detected with a streptavidin-Cy3 conjugate (Sigma, St. Louis, MO, USA) and antidigoxigenin-FITC (Roche Diagnostics, Meylan, France), respectively. Chromosomes were counterstained with DAPI (4′,6-diamidino-2-phenylindole) and mounted in Vectashield (Vector Laboratories, Burlingame, CA, USA). Hybridisation signals were visualised using a Nikon Eclipse 80i epifluorescence microscope. Images were captured with a Nikon CCD camera using the Nikon 3.0 software (Nikon Instruments Europe BV, Amstelveen, The Netherlands) and processed with Photoshop 4.0 software (Adobe Systems Inc., San Jose, CA, USA).

## Results

**Fig. 1:**
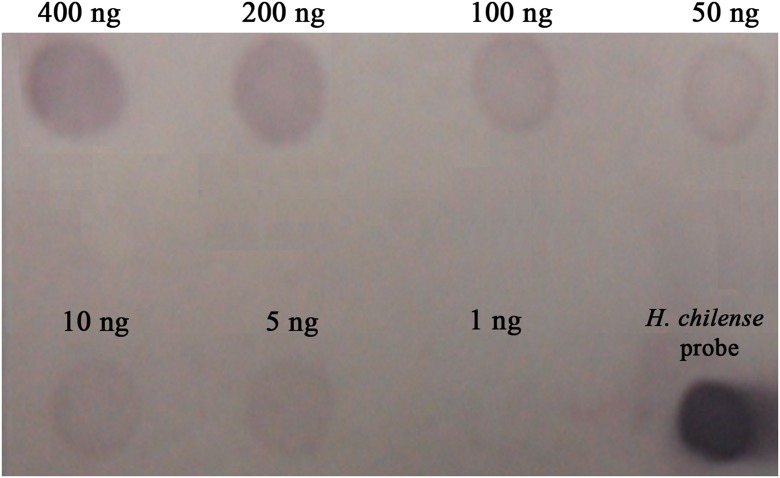
Dot-blot hybridisation experiment carried out to establish the minimum amount of total *Hordeum chilense* genomic DNA that can be detected by this technique. Biotin-labelled *H. chilense* DNA was used as a probe. Several *H. chilense* amounts of DNA ranging from 400 to 1 ng were loaded on the membrane. Positive signals were detected for all *H. chilense* amounts of DNA except when 1 ng of DNA was loaded. The minimum amount of total *H. chilense* DNA detected was 5 ng. A drop of biotin-labelled *H. chilense* DNA was used as a positive control of the procedure

With the aim of establishing the minimum amount of genomic DNA detectable in a dot-blot hybridisation, different amounts ranging from 400 ng down to 5 ng of total genomic DNA from *H. chilense* were loaded on a membrane and were hybridised using biotin-labelled total *H. chilense* genomic DNA as a probe. The dot-blot assay showed positive signals for all dots except when 1 ng of the total genomic DNA was loaded, revealing that the minimum amount of genomic DNA which is possible to detect using this technique was as little as 5 ng (Fig. 1).

To unequivocally detect *H. chilense* DNA in the background of the wheat genome and minimise wheat unspecific hybridisation signals due to common repetitive sequences between wheat and *H. chilense*, the total wheat genomic DNA was used as blocking DNA in the dot-blot experiments. Thus, it was necessary to establish the probe/blocking DNA ratio firstly to avoid false positives. A dot-blot experiment was carried out by loading two drops of 100 ng of *H. chilense* and wheat DNA, respectively, on three different membranes, which were simultaneously incubated with a different ratio of the *H. chilense* probe/blocking wheat genomic DNA in the hybridisation mixture (1:100, 1:200 and 1:300). The drop of *H. chilense* genomic DNA was used as a positive control in each membrane. We found that only when the wheat blocking DNA was 300 times more abundant than the *H. chilense* probe in the hybridisation buffer did the dot containing the wheat genomic DNA remain negative (data not shown). This meant that the ratio between the probe and the blocking DNA should be 1:300 in order to avoid false positives, due to the presence of common *H. chilense* repetitive sequences in wheat, when screening *H. chilense* genetic introgressions in the wheat background.

**Fig. 2:**
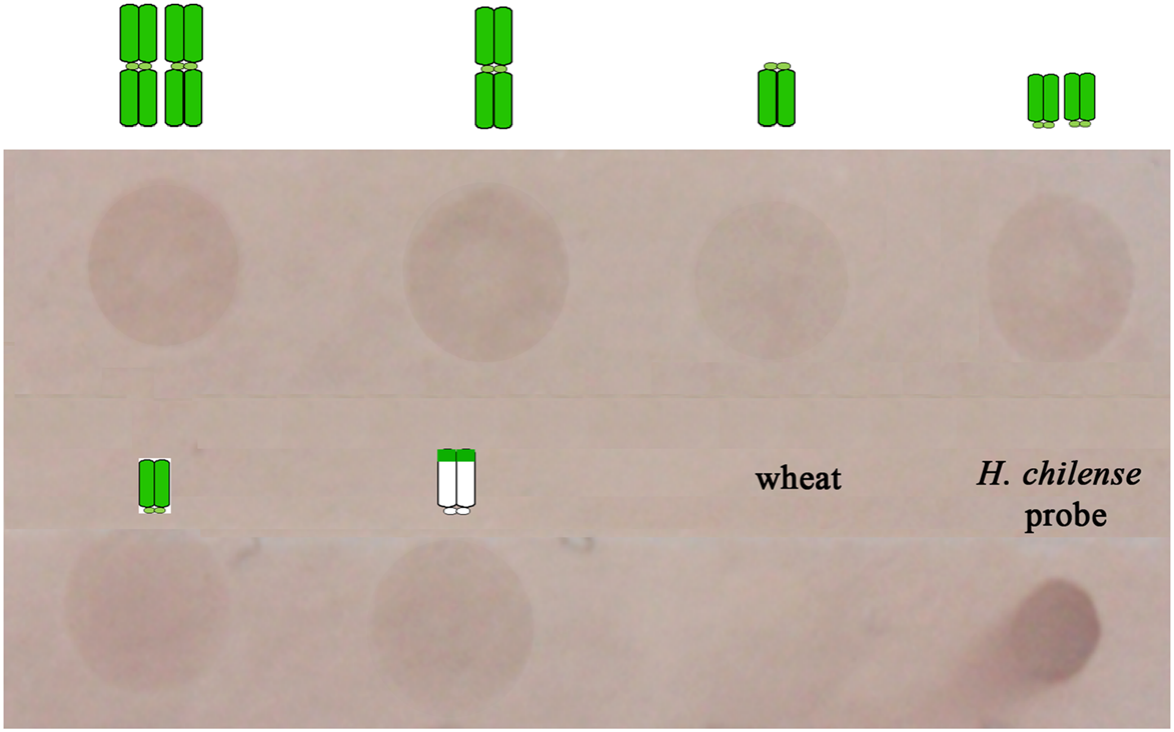
Dot-blot hybridisation assay in *Hordeum chilense* addition lines in bread wheat using biotin-labelled *H. chilense* DNA as a probe. Chromosomes in *green* represent the number of copies of the *H. chilense* chromosomes in the wheat background for each substitution line. Two hundred nanograms of total genomic DNA were loaded per sample. From *up left* to *down right*: 4**H** ^**ch**^ disomic addition line (two copies of *H. chilense* chromosomes); (4**B**) 4**H** ^**ch**^ monosomic substitution line (one copy of a *H. chilense* chromosome); 7**H** ^**ch**^L monotelosomic addition line in wheat (one copy of one *H. chilense* chromosome arm); 6**H** ^**ch**^S ditelosomic addition line in wheat (two copies of one *H. chilense* chromosome arm); 6**H** ^**ch**^S monotelosomic addition line in wheat (one copy of one *H. chilense* chromosome arm) and wheat line carrying a distal 4**H** ^**ch**^L segment (one copy of the distal region of the *H. chilense* chromosome). Positive signals were revealed for all samples. Wheat DNA was used as a negative control. A drop of biotin-labelled *H. chilense* DNA was used as a positive control of the procedure

**Fig. 3:**
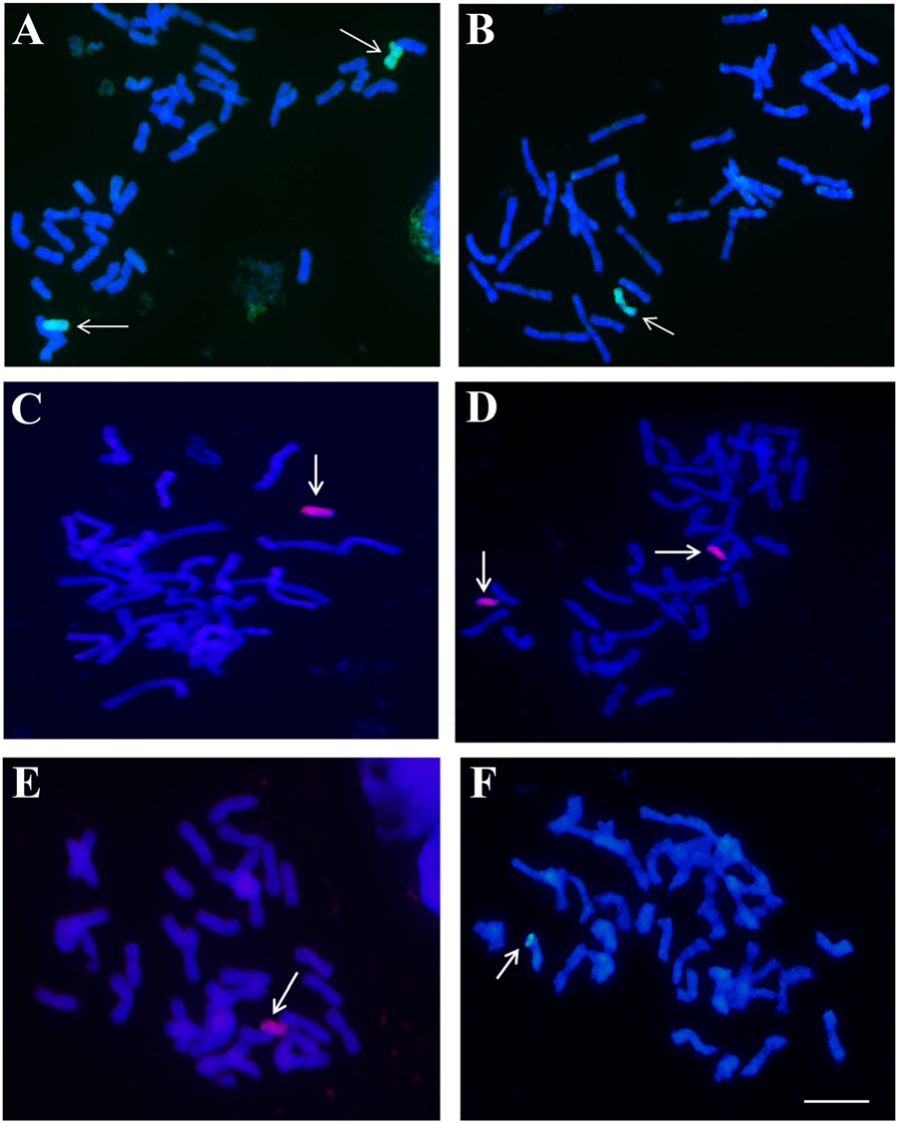
Genomic in situ hybridisation in *Hordeum chilense* introgression lines in bread wheat using biotin and digoxigenin-labelled *H. chilense* genomic DNA as probes, detected with streptavidin-Cy3 conjugates (*red*) and antidigoxigenin-FITC (*green*), respectively. **a** Chromosome 4**H** ^**ch**^ disomic addition line; **b** (4**B**)4**H** ^**ch**^ monosomic substitution line; **c** monotelosomic line for chromosome 7**H** ^**ch**^L; **d** 6**H** ^**ch**^S ditelosomic line; **e** 6**H** ^**ch**^S monotelosomic line; **f** wheat line carrying one copy of a distal 4**H** ^**ch**^L segment on 4**D** chromosome. *Scale bar* represents 10 μm for all panels

Once we determined the minimum amount of total genomic DNA detectable by this method and the ratio of the probe and the blocking DNA to avoid false positives, we tried to determine the sensitivity of this technique when the *H. chilense* genomic DNA was diluted in the background of bread wheat genome. Thus, genomic DNA from different wheat lines carrying different *H. chilense* chromosome introgressions of different sizes (disomic and monosomic *H. chilense* addition lines in bread wheat, monotelosomic and ditelosomic *H. chilense* addition lines and the wheat line carrying one copy of a distal fragment *H. chilense* chromosome 4) were loaded on a membrane. In fact, these wheat lines were chosen as representatives of *H. chilense* introgression lines that can usually be obtained in a breeding programme when different genetic crosses between, for example, wheat and *H. chilense* addition lines in wheat have been carried out. Dot-blot hybridisation results showed that it was possible to detect all these *H. chilense* genomic introgressions in the background of hexaploid wheat (Fig. 2). Moreover, the size of the *H. chilense* did not seem to be a limiting factor in this experiment to identify wheat plants carrying *H. chilense* genomic introgressions that represents at least 1/10 of a wheat chromosome, among 42 wheat chromosomes, which means approximately a 1/420 dilution of the *H. chilense* DNA in the wheat background (Fig. 2). The results obtained in the dot-blot analysis were confirmed by in situ hybridisation experiments developed in somatic cells in metaphase from the same wheat lines carrying one or two copies of an *H. chilense* chromosome, a monotelosomic or ditelosomic *H. chilense* chromosome or a distal small *H. chilense* chromosome segment in the wheat background (Fig. 3).

**Fig. 4:**
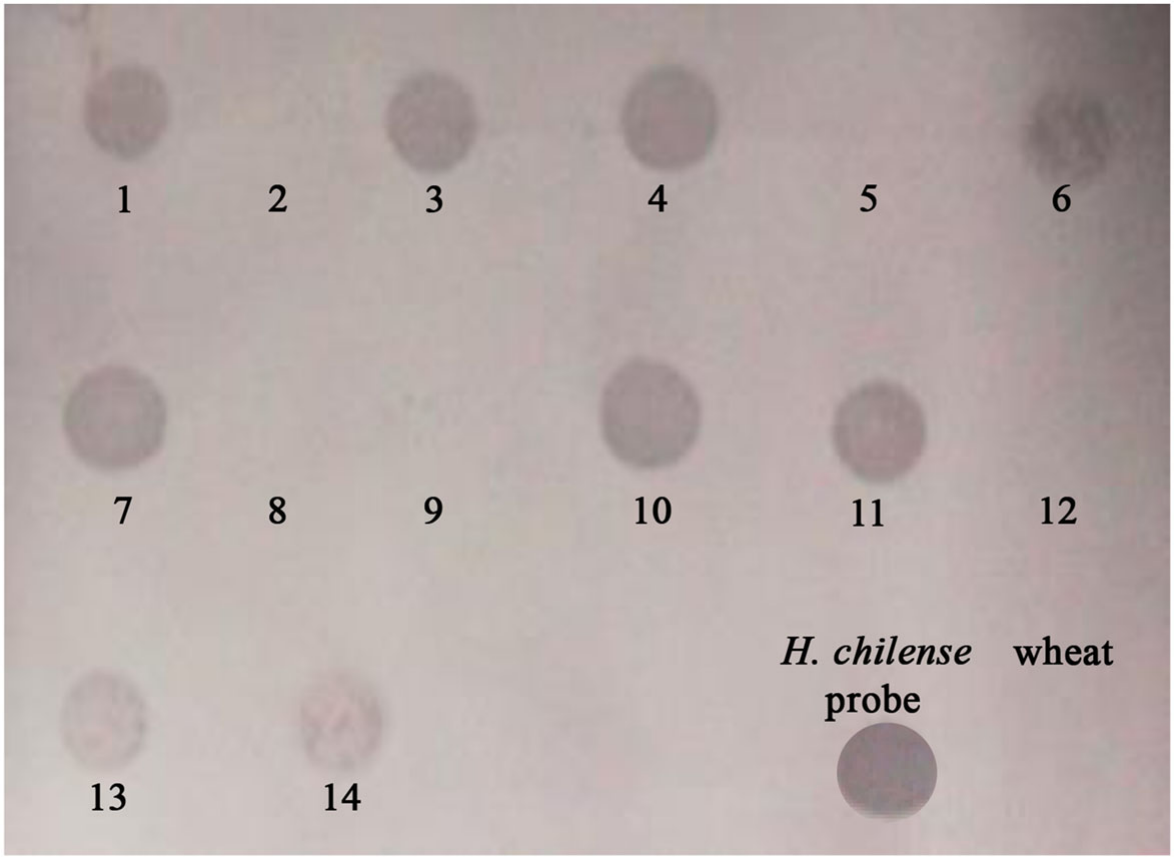
Dot-blot hybridisation in the descendence of a genetic cross between the (4**B**)4**H** ^**ch**^ monosomic substitution line and the wheat *ph1b* mutant. Two hundred nanograms of total genomic DNA from 15 plants were loaded on the membrane. Positive signals corresponded to those plants carrying *Hordeum chilense* genetic introgressions in the wheat background. A drop of biotin-labelled *H. chilense* DNA and total wheat genomic DNA were used as positive and negative controls, respectively

Once we demonstrated that the dot-blot analysis could be used to detect *H. chilense* chromosome introgressions in the wheat background, we developed the assay in the descendence of a genetic cross between the (4**B**)4**H** ^**ch**^ monosomic substitution line and the *ph1b* wheat mutant line to detect those plants carrying an *H. chilense* chromosome introgression (Fig. 4). DNA from 15 different plants were loaded on the membrane and hybridised with the *H. chilense* DNA as probe. Nine positive signals were obtained, suggesting that these plants could carry an *H. chilense* introgression in the wheat background. Genomic in situ hybridisation was performed on these 15 lines, and results did confirm the dot-blot analysis (data not shown). Positive results corresponded to wheat lines carrying *H. chilense* introgressions and negative results corresponded to wheat lines with no *H. chilense* introgressions. The method cannot inform whether it is as a full copy of one or two chromosomes or smaller genomic introgressions as the result of chromosome translocations or interspecific recombination between wheat and *H. chilense* chromosomes, but it can be used as a quick initial screening to target only those plants carrying putative *H. chilense* introgressions, which will be further analysed by in situ hybridisation, reducing the time and effort of a more detailed analysis, performed only on the desirable plants.

**Fig. 5:**
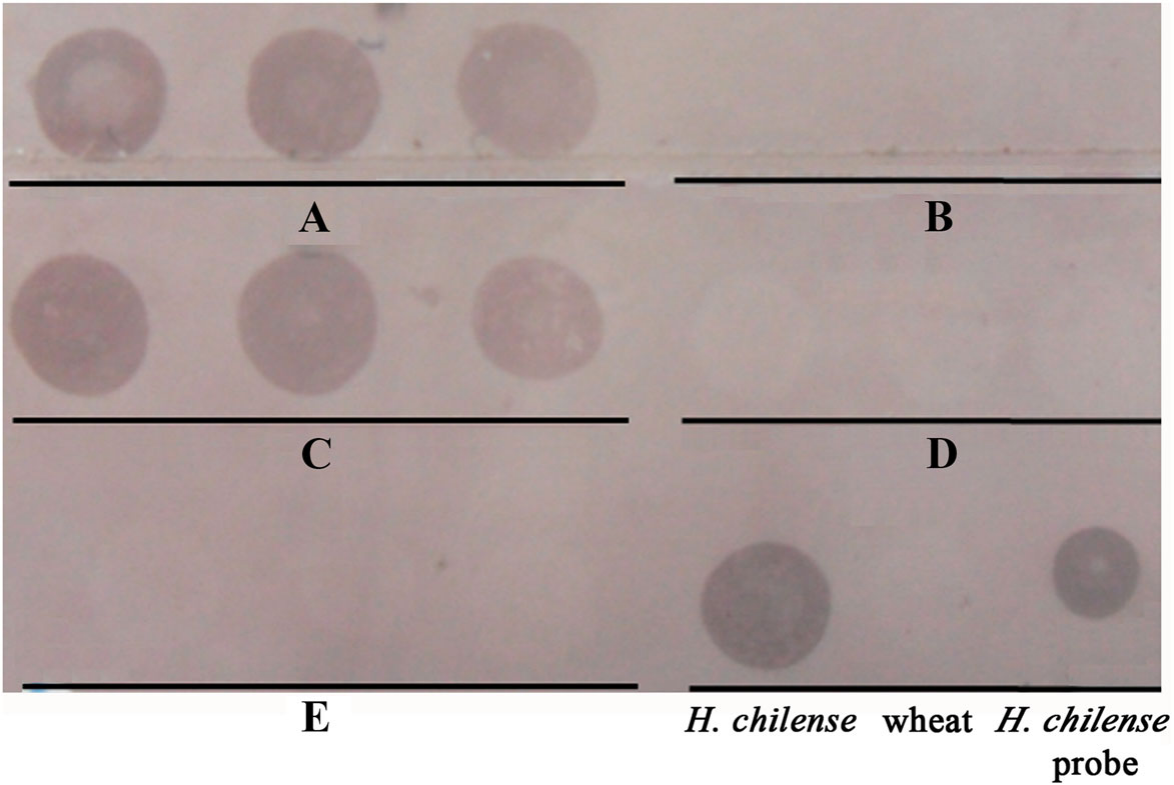
Reproducibility of the dot-blot assay for the detection of *H. chilense* genomic introgressions in the descendants of a genetic cross between a (4**B**)4**H** ^**ch**^ monosomic substitution line and the wheat *ph1b* mutant. Five different wheat lines were tested. Two hundreds nanograms of DNA from three equivalent *H. chilense* introgression lines (independent biological replicates) were loaded per sample. *Lines A* and *C* corresponded to two wheat plants carrying *H. chilense* genetic introgressions; *B*, *D* and *E* corresponded to wheat lines with no *H. chilense* chromosome introgressions. The results from the three replicates from each wheat line were consistent and the three of them were either positive or negative depending on the presence or absence of *H. chilense* introgressions in wheat. Total *H. chilense* DNA was loaded as a positive control. In addition, drops of biotin-labelled *H. chilense* DNA and total wheat genomic DNA were also used as positive and negative controls, respectively

The reproducibility of the dot-blot hybridisation for the detection of *H. chilense* DNA in the background of the bread wheat was also tested. A dot-blot experiment was carried out by loading on the membrane total genomic DNA from three equivalent plants (biological replicates) of five wheat lines from the descendants of a genetic cross between the (4**B**)4**H** ^**ch**^ monosomic substitution lines and the wheat *ph1b* mutant. Two hundred nanogrammes of the total genomic DNA were loaded per sample. Positive signals were successfully detected in the three biological replicates carrying the same *H. chilense* chromosome introgression while negative signals were consistent for the three replicates of each wheat line with no *H. chilense* genetic introgressions (Fig. 5). In addition, an equivalent experiment was repeated (technical replicate) and the observations were confirmed (data not shown). Thus, results indicated that the dot-blot hybridisation assay is a robust and reproducible method to detect *H. chilense* genomic introgressions in the wheat background easily.

**Fig. 6:**
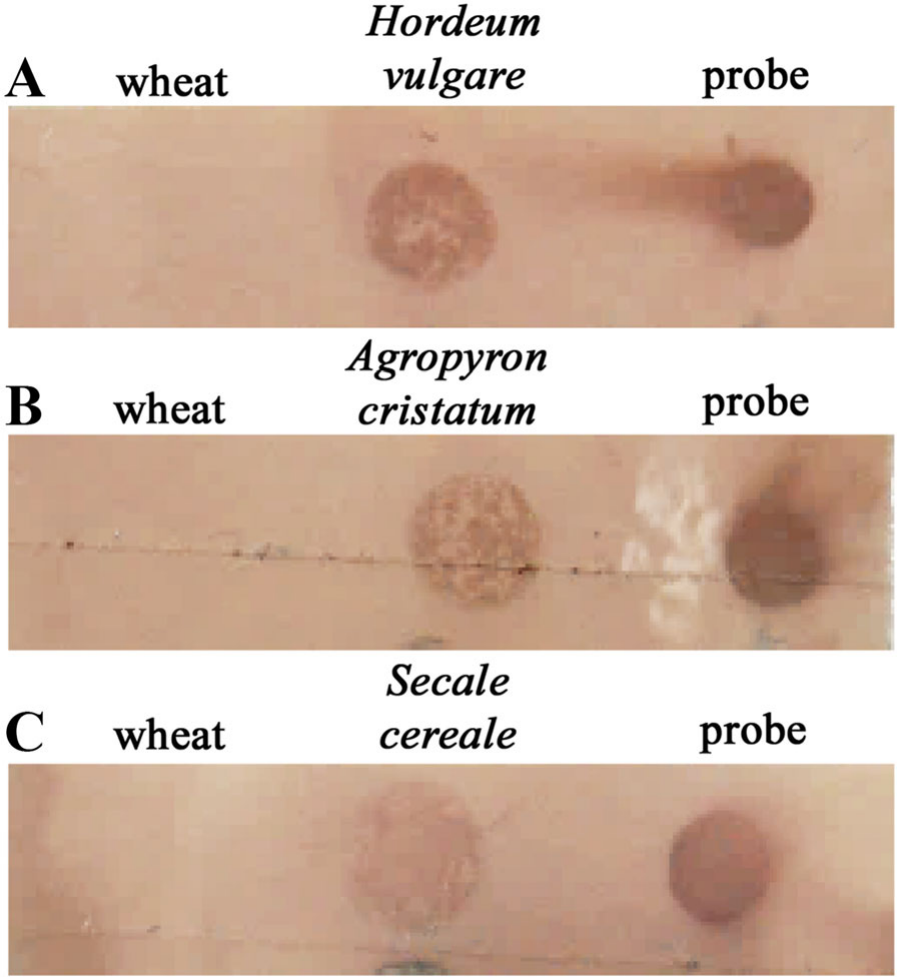
Dot-blot hybridisation experiments in the wheat relatives *Hordeum vulgare* (**a**), *Agropyron cristatum* (**b**) and *Secale cereale* (**c**). Two hundred nanogrammes of total genomic DNA from each species were loaded on each membrane. Drops of biotin-labelled *H. vulgare*, *A. cristatum* and *S. cereale* DNA were used as positive controls in each experiment. Total wheat genomic DNA was always used as a negative control. *H. vulgare*, *A. cristatum* and *S. cereale* were successfully detected by dot-blot hybridisation, and no signals were detected for the wheat DNA samples

Finally, the dot-blot assay was also validated to detect the DNA from other wheat relative species such as *H. vulgare*, *A. cristatum* and *S. cereale*. Thus, dot-blot hybridisation experiments were developed similarly to the ones described here for the detection of *H. chilense* genetic introgressions in the wheat background. The total *H. vulgare* genomic DNA was loaded in a membrane and detected in a dot-blot hybridisation assay using the total *H. vulgare* genomic DNA as a probe (Fig. 6a). The wheat genomic DNA was also loaded to be used as a negative control of the dot-blot in situ hybridisation. Similarly, this approach allowed the detection of *A. cristatum* and *S. cereale* genomic DNA (Fig. 6b, c, respectively). The ratio of each *H. vulgare, A. cristatum* and *S. cereale* total genomic probes and the wheat blocking DNA for these experiments was similar (1:300) to the one used for the detection of *H. chilense* genomic introgressions. No signals were detected for the wheat-negative control in any case. These results revealed that dot-blot hybridisation could be also used to easily screen *H. vulgare*, *A. cristatum* and *S. cereale* genomic introgressions in the wheat background.

## Discussion

Dot-blot hybridisation assay has been revealed as a user-friendly assay that can be used as a routine tool for a rapid screening of genetic introgressions from related species in a wheat population. The method is especially useful when alien genetic introgressions are random and the screening using molecular markers would be a challenge or molecular markers cannot be associated to the introgressed segment. The total genomic DNA from *H. chilense* was labelled indistinctly with biotin-11-dUTP or digoxigenin-11-dUTP and used as probes for in situ hybridisation. Probes were detected with Cy3 or FITC, respectively. Biotin labelling was routinely used to detect lower amounts of exotic *H. chilense* DNA in the wheat background in dot-blot experiments, although digoxigenin could also be used for DNA labelling.

Nowadays, there are several techniques available for labelling and detecting alien introgression in the wheat background, including C-banding, molecular markers or in situ hybridisation. Although molecular markers and in situ hybridisation are useful tools to select the desirable plant material carrying genomic introgressions (Forster et al. 2000; Prieto et al. 2001), both techniques are high cost and time consuming. It has been estimated the high cost of labour, reagents, antibodies and fluorochromes for manual in situ hybridisation and their increment with the number of samples, due to the time required for sample manipulation and the increment of reagents to perform the protocol of a high number of samples even when some steps could be automated (Zanatta et al. 2015). It is worthy to take into account that the longest and consequently, the most expensive step during the in situ hybridisation methodology is the preparation of chromosome spreads, particularly when plants are the targets, which is not even included in estimation costs studies, due to the difficulty and variability in time of obtaining good chromosome spreads suitable for in situ hybridisation experiments. Thus, the methodology described here can reduce considerably the cost of the screening of chromosome introgressions not only because of the lower cost of the protocol itself (less reagents and no fluorochromes are needed) but also for the lower number of plants required for analysis by in situ hybridisation. On the other hand, several DNA extraction methods have been optimised for molecular marker analysis, which can also be cheaper than in situ hybridisation (Xin and Chen 2012; He et al. 2014). However, the use of molecular markers can be limited when small fragments of related species are achieved in a crop such as bread wheat, mainly because genetic maps of related species such as *H. chilense* are not saturated (Hernández et al. 2001). In addition, the use of molecular markers is based on a previous knowledge of the exact chromosome introgression to choose the most suitable markers, but can be useless when the chromosome or chromosome segment from the related species involved in recombination with the wheat chromosomes are unknown, resulting in random chromosome introgressions from the related species in the wheat background. The detection of genomic introgressions using molecular markers can also be limited by the high level of synteny among wheat and related species (Moore et al. 1995; Salse and Feuillet 2007), making it difficult to find specific markers for the introgressed chromosome fragment in the wheat background. For example, the difficulty to detect alien genomic DNA in the wheat background was already reported in *Thinopyrum intermedium*-wheat recombinants since there were not enough molecular markers to determine the presence of *T. intermedium* in wheat (Qi et al. 2007). In fact, only 9 out of 16,000 EST markers were polymorphic to define the *T. intermedium* introgression regions in the wheat background (Qi et al. 2007). In contrast, in the dot-blot experiments reported here, it was possible to unequivocally distinguish *H. chilense* genetic introgressions in the wheat background due to the optimisation of the blocking DNA concentration, which was a key step caused by the high presence of common repeat DNA sequences (>75%) in cereals (Flavell and Smith 1976; Flavell et al. 1977; Bedbrook et al. 1980; Choulet et al. 2010; Brenchley et al. 2012). Several concentrations of wheat blocking DNA were evaluated in an attempt to enhance the sensitivity required in the dot-blot hybridisation to clearly detect *H. chilense* genetic introgressions in the wheat background. The optimal probe/blocking DNA ratio in the hybridisation mixture to avoid false positives (1:300) was similar to the one described by Sanchez-Moran et al. (2001), where a rate of 1:2:300 was used for A genome probe/D genome probe/B genome as blocking DNA, respectively, in the hybridisation mixture to successfully discriminate the A, B and D wheat genomes in cytogenetic experiments. In addition, some other probe/blocking DNA ratios (1:100 and 1:200) were tested in the hybridisation mixture, but in both cases, results were not convincing and false positives were detected.

On the other hand, in situ hybridisation is the most efficient and most accurate technique to estimate the genetic composition in plants or identify and characterise chromosome translocations in plants (Le et al. 1989; Schwarzacher et al. 1989; Jiang and Gill 1994) but the cost of in situ hybridisation experiments is high and requires more time and higher expertise to prepare and process the samples as it is a technically more complicated methodology. Thus, in situ hybridisation is not the most appropriate tool for screening a high number of plants from a segregating population. Therefore, in this work, we describe a more convenient and efficient method which has been extensively used for years in other plant applications (Owens and Diener 1981; Besse et al. 1997; Liu et al. 2007; Tonosaki and Nishio 2010; Vassilakos et al. 2012; Azza and Eman 2016) and does not require high qualification to prepare the samples and perform the experiments in order to identify, for example, those plants carrying exotic chromosome introgressions in a segregation population.

In fact, dot-blot hybridisation can be very useful in wheat breeding programmes when the manipulation of chromosome associations between wheat chromosomes and those from related wheat species used as genetic donors is carried out in the absence of the *Ph1* locus (Sears 1977), which has been widely used to transfer useful genes from wild relatives into wheat to obtain resistance to biotic and abiotic stresses (Friebe et al. 1996; Xin et al. 2001; Mullan et al. 2009). The use of either only molecular markers or in situ hybridisation would be high cost and difficult in this context to locate small alien genetic introgressions, whereas dot-blot hybridisation would facilitate the screening of those chromosome introgressions, being faster, cheaper and more reliable.

Although there are a high proportion of repeated sequences in cereals, the sensitivity and reliability of the molecular hybridisation assay described here introduce a basic and simple method to detect the presence of chromosome introgressions from relative species in the wheat background. Moreover, the detection of other wheat relative species such as *H. vulgare*, *S. cereale* and *A. cristatum* in the wheat background was also possible using the dot-blot hybridisation assay. As a result, dot-blot hybridisation has been revealed as an efficient and user-friendly method for the screening and selection of plants carrying small random genomic introgressions from an alien species into the wheat germplasm, to be further fully characterised by the use of other approaches such as molecular markers or in situ hybridisation.
